# Editorial: Breaking the cycle: exploring the interplay of conflict, hunger, poverty, and food insecurity in Africa and other regions, and implications for policy

**DOI:** 10.3389/fnut.2026.1963113

**Published:** 2026-09-14

**Authors:** Olutosin Ademola Otekunrin, Temitope Oluwaseun Ojo, Barbara Sawicka, Idris Akanbi Ayinde

**Affiliations:** 1 Department of Agribusiness, Federal University of Agriculture and Development Studies, Iragbiji (FUADSI), Iragbiji, Nigeria; 2 Innovation Lab for Policy Leadership in Agriculture and Food Security (PiLAF), University of Ibadan, Ibadan, Nigeria; 3 Disaster Management Training and Education Centre for Africa, University of the Free State, Bloemfontein, South Africa; 4 Department of Business and Social Sciences, Faculty of Agriculture, Dalhousie University, Halifax, NS, Canada; 5 Department of Plant, Food and Environmental Sciences, Faculty of Agriculture, Dalhousie University, Halifax, NS, Canada; 6 Department of Plant Production Technology and Commodities Science, University of Life Science in Lublin, Lublin, Poland; 7 Department of Agricultural Economics and Farm Management, Federal University of Agriculture, Abeokuta (FUNAAB), Abeokuta, Nigeria

**Keywords:** developing countries, extreme poverty, food security, humanitarian aid, violent conflict

## 1 Introduction

Attaining the United Nations targets for Sustainable Development Goal 1 (No Poverty) and 2 (Zero Hunger) in less than 5 years from now (2030), have remained a challenging task for low- and middle-income countries, especially Africa, which hosts the largest number of people (309 million) facing hunger globally in 2025. It was reported that one out of every five people (20%) in Africa face hunger in 2025, compared to 6% of the population in Asia, 4.8% in Latin America and the Caribbean, 8% in Oceania and below 2.5% in Northern America and Europe (1).

In recent time, the global agrifood system is operating within a complicated “interlocking crises”—a situation where cross-border conflicts, climate fluctuations, and macroeconomic volatility converge to create cascading vulnerabilities (2–5).

At the heart of this crisis lies the vicious, self-reinforcing feedback loop: the damaging interaction of regional conflict, chronic hunger, entrenched poverty, and critical food insecurity. Far from operating as isolated socioeconomic disruptions, these crises operate as interconnected, bidirectional drivers of vulnerability. While poverty and conflict drive disruptions to the physical availability and economic accessibility of food, these shocks ultimately result in food insecurity, characterized by severe constraints on food availability and physical and economic access to food, thereby translating macro-level shocks into chronic hunger and malnutrition (5–8).

In vulnerable regions, conflict serves as the key disruptor of agricultural value chains. It undermines agricultural production by limiting access to farmland, severing agricultural input supply chains, and destroying critical physical infrastructure. Furthermore, chronic food insecurity and systemic rural poverty are not just passive outcomes of unpredictability; they act as catalysts for social unrest, fueling civil conflict over scarce natural resources such as arable land and water resources.

In Nigeria and other parts of West Africa, these enduring challenges are deeply rooted in socioeconomic inequalities, market fragmentation, and limited institutional support for smallholder farmers, who account for the vast majority of the region's food production (8–10).

Furthermore, previous studies have highlighted the slow uptake of modern, intensive agricultural practices, combined with the underutilization of highly climate-resilient, nutrient-dense “orphan crops” like taro (*Colocasia esculenta*), has left rural food systems highly vulnerable to external shocks (11). Advancing these vulnerable, subsistence-based farming systems into commercially integrated, value-added agricultural value chains indicates a critical but historically overlooked pathway to enhancing both dietary resilience and household economic wellbeing.

This Research Topic (RT) was conceptualized to map, deconstruct, and propose empirical, policy-relevant solutions to this conflict-hunger-poverty-food insecurity nexus. This Research Topic brought together 19 articles from diverse geographical contexts, methodological approaches, and disciplinary perspectives, combining agronomy, agricultural economics, public health nutrition, and policy analysis to chart a resilient pathway toward sustainable food systems.

## 2 Conceptual framework: systemic feedback loops of vulnerability

To understand the structural unity of the 19 articles in this volume, we must establish a clear Systems-Level Conceptual Framework. The interactions among conflict, hunger, poverty, and food insecurity are non-linear, functioning as a self-reinforcing feedback system of interconnected feedback loops.

The systemic complexity of these interactions is illustrated in the conceptual framework presented in Figure 1, which maps the reinforcing feedback loops that either trap households in a self-perpetuating “*cycle of vulnerability*” or through targeted policy interventions, shift them onto a “*virtuous cycle of resilience*.” This conceptual framework was similar to the study of Tidball et al. (12) on community-led reforestation.

**Figure 1:**
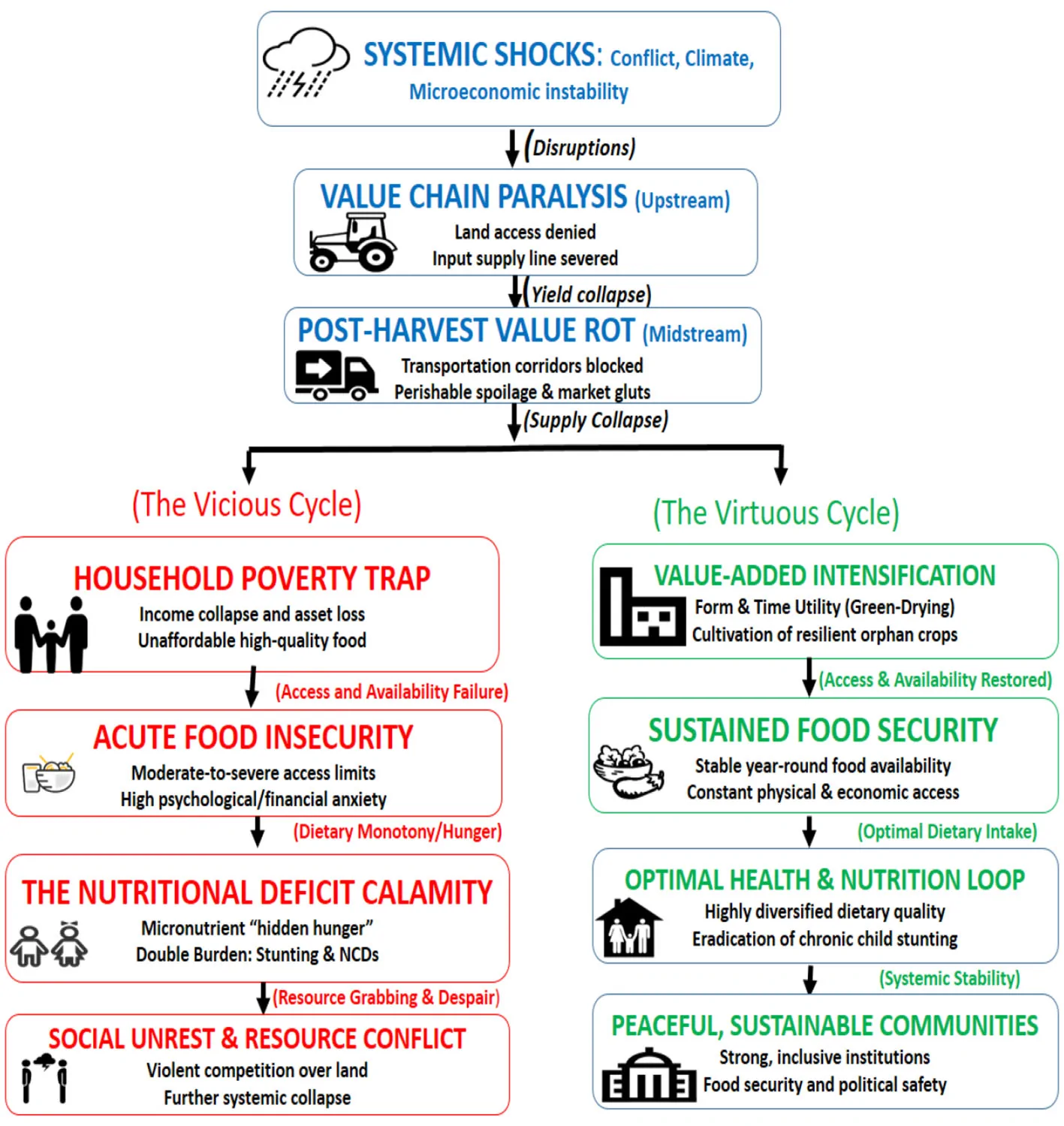
The systems-level feedback loop of the conflict-hunger-poverty-food insecurity nexus. Source: Authors' own conceptualization.

As depicted in the conceptual model, agricultural value chain paralysis and post-harvest losses do not directly translate into clinical malnutrition; rather, they must first pass through the critical intermediary stage of severe food insecurity before progressing to clinical malnutrition.

Under the vicious cycle, the supply collapse of essential food supplies drives households into moderate-to-severe food insecurity, characterized by heightened anxiety over food access and an involuntary reliance on low-cost, nutrient-poor staple diets.

Conversely, the resilience-oriented intervention pathway disrupts this vicious cycle by reducing systemic risks and weakening the reinforcing feedback loops that drive household vulnerability. By integrating value-added intensification with post-harvest dehydration (green-drying), the system enhances food security by ensuring sustained year-round physical and economic access to safe and nutritious foods, thereby promoting optimal dietary health, nutritional security, and community resilience.

## 3 Thematic synthesis of the Research Topic

To provide a logical and structured reading of the 19 articles, this volume is divided into distinct, overlapping themes.

• Pillar 1 (Articles 1–9): focuses on the macro-disruptions of conflict, spatial market mismatches, agricultural production shocks, and early-stage supply chain vulnerabilities.

From the East African perspective, Omay et al. with the title: “*Insights into the determinants influencing food security in the IGAD region of Eastern Africa*,” examined how food security in the Intergovernmental Authority on Development (IGAD) region of East Africa was influenced by complex interaction of both climatic and non-climatic factors such as severe weather, land management, market mechanisms, and macroeconomic volatility. Authors found out that hydroclimatic (dry-wet) extremes considerably influenced food security status while unfair trade practices and inadequate agricultural funding across IGAD nations contributed to escalating food costs and undernourishment. The study emphasized the urgent need for intersectoral and geographically aligned strategies to promote food security.

Evidence from Ethiopia shows how structural pressures are experienced through household diets. Jisso et al. examined household food security and maternal dietary diversity in rural Gedeo, linking dietary diversity with wealth, maternal education, pregnancy status, and food-security conditions. Their contribution shifts attention from whether food is available to whether women can obtain varied, nutritious diets. Policies centered only on staple production may overlook micronutrient inadequacy and unequal access within households. Maternal literacy, women's income opportunities, nutrition education, and social protection should be central components of food-security policy.

Mulatu et al. deepen this maternal-nutrition perspective by assessing dietary practices among lactating mothers in Dangila District, Ethiopia. Although many women achieved adequate dietary diversity, only one-third demonstrated good dietary practices, while food insecurity affected most respondents. Poor practices were associated with daily labor, larger families, short birth intervals, and food insecurity. Nutritional knowledge alone cannot overcome economic pressure, time constraints, and competing household needs. The study supports practical dietary counseling within maternal health services, stronger family planning, improved livelihood opportunities, and coordination among health, agriculture, and social-welfare programs.

Jambo et al. explore a nutrition-sensitive pathway through the adoption of quality protein maize in southern Ethiopia. The crop can improve access to amino acids where maize is a staple, but its benefits depend on farmers' ability and willingness to cultivate it. Seed access, land size, income, education, extension contact, field-day participation, and livelihood diversification influenced adoption. The findings show that nutritionally improved crops cannot succeed through varietal development alone. Reliable seed systems, practical extension, farmer demonstrations, finance, communication, and markets are needed to make adoption feasible for smallholders.

Bila et al. in their study titled “*Assessment of farmer's knowledge and attitudes toward fungi and mycotoxin contamination in staple crops in southern Mozambique*” assessed the understanding and views of 180 smallholder farmers about fungi and mycotoxin contamination of food crops, food loss, and income in Southern Mozambique. The study revealed that almost all the farmers (98%) showed insufficient level of understanding about fungi and mycotoxins contamination in food crops and agricultural products. The study highlighted the urgent need for training, affordable drying and storage technologies, surveillance, stronger food-safety regulation, and extension services for farmers that translate knowledge into practical action.

In the study of Lukwa et al. titled “*Impact of COVID-19 on household hunger and socio-economic inequality in South Africa: a comparative analysis using NID-CRAM (2020–2021) and NFNSS 2022*” compared household hunger in the period of severe pandemic and early recovery phase and investigated how social gaps in food security evolved. Authors revealed that notable provinces in South Africa such as Northern Cape, Free State, and North West continued to be fairly food insecure during this periods. The study highlighted the need for sustained structural policy measures that promote social inclusion and fix regional and infrastructural deficits to enhance resilient food security.

Cao et al. in their study titled “*Environmental sustainability and nutritional quality: addressing global food and nutrition security in a post-pandemic world*” employed the IMPACT model, alongside climate forecasts, field trials, among others to explore international food supply. The authors emphasized the need for sustainable agriculture and climate adaptation to promote food security in an ever-evolving world dynamics.

Ojo et al. examine the bridge between innovation and farmers by studying extension agents' attitudes and participation in disseminating climate-smart agricultural practices in North-Central Nigeria. More than half held unfavorable attitudes, while participation in communicating water- and crop-management options was limited. Training, experience, characteristics, and attitudes influenced engagement. The study shows that innovations do not spread simply because they are technically effective. Extension personnel require training, logistical support, motivation, workloads, and recognition to improve farmers' access to climate information, water management, insurance, improved varieties, integrated pest management, and diversified production.

Niu et al. in their study titled “*Definition of the traditional African diet: a scoping review*” systematically investigated the definition of the traditional African diet (TrAfDi) through the lens of a scoping review. Authors reported that out of 45 included articles, cereals, pulses, seeds and nuts, vegetables and their products are among the food groups that defined the TrAfDi. The study presented a preliminary evidence on what formed a TrAfDi and revealed attributes of its indigenous qualities which are germane to designing public health strategies to address hunger crisis, overweight, and non-infectious diseases in Africa.

- Pillar 2 (Articles 10–19): shifts the analytical lens to micro-level consumer sensory preferences, clinical and household nutrition, economic disempowerment, climate-smart agriculture (CSA) adoptions, and regional socio-demographic disparities.

The study of Tenza et al. titled “*Consumer sensory assessments of chicken breeds: the role of location, age, and gender in resource-poor communities for food security and sustainable livelihoods*” investigated the impact of location, age, and gender of consumers on sensory attributes of 120 village chickens breeds and broiler chickens in under-resourced communities. Authors found out that the color scale of both male and female chickens were fairly the same (5.02–5.57) and emphasized the impact of location, age, and gender of consumers in sensory qualities on the consumer sensory evaluations.

To understand the macro-level drivers of agricultural disruption in West Africa, Diori et al. in their study titled “*Protracted violence and insecurity: a quantitative analysis of persistent drivers of rising conflict in the Sahel*” deployed a robust econometric model to investigate the underlying drivers of escalating, prolonged conflict across the Sahel. By integrating geo-referenced historical conflict data from the armed conflict location and event data (ACLED) database with micro-level socioeconomic measures, the authors mapped the causal pathways of agricultural vulnerability. Their quantitative analysis demonstrated that while climatic variability, such as climate-induced rainfall shocks and rangeland degradation, serve as threat multipliers, they are not the underlying drivers of violence. Instead, the structural drivers of conflict in the Sahel are institutional and political, marked by structural youth unemployment, perceived political and socioeconomic exclusion, fragile rule of law, insecure land tenure systems for pastoralist and crop-producing communities. This research establishes that predominantly military-oriented security approaches are insufficient in building resilient food systems in fragile settings, highlighting that policymakers must instead prioritize building resilient land-use conflict resolution institutions, strengthening rural technical and vocational education and training, and supporting youth-led agribusiness value chains to provide viable economic opportunities that reduce vulnerability to conflict participation.

Operating within Mali's fragile and conflict-affected ecological and sociopolitical context, Kangas et al. developed a robust “Theory of Change” (ToC) for how relapse can be reduced in Mali and propose an intervention package that is feasible for the health system to adopt with minimal external support. The researchers integrated community-based healthcare providers, mothers, and humanitarian practitioners to develop a post-intervention surveillance protocol focused on Small-Quantity Lipid-Based Nutrient Supplements (SQ-LNS). The participatory ToC systematically mapped the multidimensional operational, household, and environmental constraints that drive post-treatment relapse, confirming that the routine provision of SQ-LNS, combined with structured, community-based growth monitoring, creates a resilient post-intervention safety net that substantially lowers the likelihood of nutritional decline. This study provides a practical and adaptable framework for public health administrators, demonstrating that clinical management of malnutrition must be paired with structured, food-based post-discharge surveillance and sustainable locally sourced nutrient supplement value chains to promote long-term child survival in conflict-affected food environments.

Focusing on Nigeria's agricultural Middle Belt region, Ogunfolaju et al. in their study titled “*Determinants of food insecurity among smallholder farmers in North Central Nigeria: policy implications*” explored the household-level factors influencing food and nutrition security among smallholder farmers. The authors employed the Household Food Insecurity Access Scale (HFIAS) to measure food security status and analyzed the drivers of food insecurity using an ordered probit model regression. The econometric findings revealed the sex of the household head, marital status, level of education and other important socioeconomic factors significantly influenced food security status of the farming households. The article emphasized the need for agricultural policies in Nigeria to promote equitable access to education and extension services as a means of improving household food security among smallholder farming households.

Addressing an increasingly important public health challenge, Omokpariola et al. presents an integrated, multi-country epidemiological and food systems assessment with the title: “*Malnutrition and double burden of disease: African dilemma of non-communicable disease*.” The authors employed a narrative approach to explore the double burden of malnutrition and its relationship with non-communicable diseases (NCDs) in Africa. The study reveals a growing shift where accelerated urbanization, physically inactive lifestyle, and the proliferation of inexpensive imported ultra-processed foods in local retail markets contributed to an epidemiological transition. Consequently, cardiovascular diseases, hypertension, and type 2 diabetes are rising rapidly among populations residing in urban and peri-urban settings, while the rural households continue to struggle with chronic stunting. The study calls for fundamental shift in African food and agricultural policies, arguing that national development plans must move beyond the narrow objective of maximizing the production of starchy staple crops instead implement fiscal and regulatory incentives that promote the development of local value chains to encourage the availability and affordability of nutrient-dense, diverse, and minimally processed local foods.

In exploring the ecological-security nexus in Nigeria, Edewor and Ogbe conduct a structured qualitative and quantitative review of how climate-affected environmental degradation triggers violent disputes over access to natural resource, particularly farmer-herder conflicts, thereby contributing to national food insecurity. The study demonstrates how rapid desertification, soil erosion, and drying water basins in northern Nigeria contributes to southward pastoralists migration, thereby intensifying violent conflicts over customary land-use rights with farming communities. To enhance the resilience of food value chains, the authors call for a dual-policy response that integrates climate adaptation with peacebuilding, recommending that governments invest in green security infrastructure, establish ranching systems to reduce open grazing, and strengthen community-led joint peace committees.

Khumalo et al., in their article titled “*The association between climate-smart agriculture practices adoption and farm income and wealth of small-scale urban crop farmers in eThekwini municipality, with implications for food and nutrition security*” investigated the association between Climate-Smart Agricultural (CSA) practices adopted by smallholder urban crop farmers and farm income and household wealth in the eThekwini Municipality. The study employed quantitative household-level survey data and applied multiple linear regression models alongside asset index decomposition analysis to evaluate changes in farm income and household wealth. The modeling results indicated that adopting number of CSA practices was positively and significantly associated with higher household farm income and wealth accumulation. The authors emphasized the need for municipal planning frameworks to formally integrate urban agriculture, secure land-use rights for urban farmers, and provide targeted technical training in climate-smart agronomy.

Investigating the vicious cycle of poverty, malnutrition, and limited education attainment Ajayi and Oyekunle present a study titled: “*An analysis of economic disempowerment as a fundamental barrier to food insecurity and education access in Nigeria*” about household economic marginalization serves as a leading, intergenerational disadvantage to food security and access to education in Nigeria. The authors utilized household survey data of 450 respondents, exploring the impacts of insecurity and socioeconomic and political instability on food security and educational outcomes. The study analyzed trade-offs that low-income households are forced to make during periods of economic hardship, indicating that extreme income poverty compels households to adopt negative coping strategies. Farm families are compelled to prioritize low-cost, nutrient-poor starchy staples over nutritionally diverse diets to secure their basic means of subsistence, thereby exacerbating chronic micronutrient malnutrition and childhood stunting.

The authors argue that sustainable food and nutrition security interventions must be integrated with comprehensive social protection frameworks, including conditional cash transfers and school feeding programs, to improve households' socioeconomic wellbeing.

Exploring regional agricultural transformations, Akpabio et al. offer a pivotal, evidence-based assessment of the opportunities and structural barriers of expanding the adoption of Climate-Smart Agriculture (CSA) technologies to advance the achievement of SDG 2 (Zero Hunger) across the agroecological zones of West Africa. The authors conducted a systematic evidence synthesis of regional adoption data, emphasizing that while CSA practices possess offer transformative for building climate resilience, their scaling up is hindered by persistent structural constraints such as high start-up costs, weak public agricultural extension systems, and limited access to weather-indexed insurance. The study emphasized that achieving SDG 2 in West Africa requires addressing institutional bottlenecks, calling for regional bodies such as ECOWAS to establish targeted green financing mechanisms to support CSA investment in the subregion.

In conclusion, Kitole et al. in their article titled: “*Rural-urban disparities in child nutrition in Tabora, Tanzania: a socioeconomic decomposition and implications for food security policy*” employed Blinder-Oaxaca and socioeconomic decomposition analyses to investigate the determinants of the child nutritional gap across rural and urban settings in Tanzania. The authors measured child nutritional status using standard anthropometric *Z*-scores for stunting, wasting, and underweight. The decomposition analysis indicated that the gap is almost entirely explained by inequalities in maternal education, healthcare utilization, household wealth measures, and access to clean water and sanitation (WASH) infrastructure.

## 4 Conclusion

The collective evidence presented in this Research Topic emphasized that addressing the conflict-hunger-poverty-food insecurity nexus requires moving beyond fragmented, single-sector interventions. We must design and implement holistic, systems-level strategies that treat agriculture not merely as a subsistence farm-gate practice, but as a coordinated, high-tech, and risk-resilient Agribusiness System.

In conclusion, the editorial team expresses its deepest gratitude to the contributing authors for their pioneering research, and to the dedicated peer reviewers whose rigorous, constructive evaluations ensured the scientific excellence of this volume.

We also sincerely thank the editorial offices of Frontiers in Nutrition and Sustainable Food Systems for their support.

It is our profound hope that the empirical frameworks presented in this Research Topic will serve as a definitive, practical guide for policymakers, researchers, and agribusiness leaders as they collaborate to construct sustainable, food-secure, and peaceful societies worldwide.
